# Priming of Production in Maize of Volatile Organic Defence Compounds by the Natural Plant Activator *cis*-Jasmone

**DOI:** 10.1371/journal.pone.0062299

**Published:** 2013-06-26

**Authors:** Sunday Oluwafemi, Sarah Y. Dewhirst, Nathalie Veyrat, Stephen Powers, Toby J. A. Bruce, John C. Caulfield, John A. Pickett, Michael A. Birkett

**Affiliations:** 1 Department of Crop Production, Soil and Environmental Management, Bowen University, Iwo, Osun State, Nigeria; 2 Biological Chemistry and Crop Protection Department, Rothamsted Research, Harpenden, Herts., United Kingdom; 3 University of Neuchâtel, Institute of Biology, Neuchâtel, Switzerland; 4 Biomathematics and Bioinformatics Department, Rothamsted Research, Harpenden, Herts., United Kingdom; Cinvestav, Mexico

## Abstract

*cis*-Jasmone (CJ) is a natural plant product that activates defence against herbivores in model and crop plants. In this study, we investigated whether CJ could prime defence in maize, *Zea mays*, against the leafhopper, *Cicadulina storeyi*, responsible for the transmission of maize streak virus (MSV). Priming occurs when a pre-treatment, in this case CJ, increases the potency and speed of a defence response upon subsequent attack on the plant. Here, we tested insect responses to plant volatile organic compounds (VOCs) using a Y-tube olfactometer bioassay. Our initial experiments showed that, in this system, there was no significant response of the herbivore to CJ itself and no difference in response to VOCs collected from unexposed plants compared to CJ exposed plants, both without insects. VOCs were then collected from *C. storeyi*-infested maize seedlings with and without CJ pre-treatment. The bioassay revealed a significant preference by this pest for VOCs from infested seedlings without the CJ pre-treatment. A timed series of VOC collections and bioassays showed that the effect was strongest in the first 22 h of insect infestation, i.e. before the insects had themselves induced a change in VOC emission. Chemical analysis showed that treatment of maize seedlings with CJ, followed by exposure to *C. storeyi*, led to a significant increase in emission of the defensive sesquiterpenes (*E*)-(1*R*,9*S*)-caryophyllene, (*E*)-α-bergamotene, (*E*)-β-farnesene and (*E*)-4,8-dimethyl-1,3,7-nonatriene, known to act as herbivore repellents. The chemical analysis explains the behavioural effects observed in the olfactometer, as the CJ treatment caused plants to emit a blend of VOCs comprising more of the repellent components in the first 22 h of insect infestation than control plants. The speed and potency of VOC emission was increased by the CJ pre-treatment. This is the first indication that CJ can prime plants for enhanced production of defensive VOCs antagonist towards herbivores.

## Introduction

Maize, *Zea mays* L. (Poaceae), is the third major cereal crop in the world after wheat and rice, and supplies 50% of the calorific intake in sub-Saharan Africa. In some years, a farmer's entire crop can be wiped out by maize streak virus (MSV) (Geminiviridae: Mastrevirus), an endemic pathogen of native African grasses and the most important plant virus disease of maize in sub-Saharan Africa [1]. It is acquired and transmitted by nine leafhopper species (Homoptera: Cicadellidae) in the genus *Cicadulina*, in a persistent manner. Although the ability to transmit MSV varies between *Cicadulina* species, some can retain and transmit the virus throughout their life. There are five species, *C. storeyi*, *C. mbila*, *C. arachidis*, *C. ghaurii* and *C. dabrowskii*, involved in MSV transmission in Nigeria, the first two species being the most efficient and therefore causing most economic losses [2], [3]. In Nigeria, 100% infection has been recorded in some fields, while outbreaks of the disease, and subsequent economic losses, have been reported in over 20 countries [4]. The negative impact of leafhopper populations upon maize production makes the search for alternative control methods extremely urgent. The potential for exploiting semiochemicals (naturally-occurring behaviour or development-modifying chemicals) in maize/leafhopper interactions, with a view to their deployment in leafhopper control, was investigated in our earlier work [5].

Plants respond to insect herbivory by the release of blends of volatile organic compounds (VOCs) that are attractive to natural enemies of herbivores [6], [7]. This aspect of plant/insect interactions has received huge interest, not only because of the scientific interest, but also for its potential application in sustainable pest control strategies. The role of induced VOCs influencing defence pathways in neighbouring undamaged plants has been discussed and described previously [8], [9]. Whilst induced defence VOCs can immediately induce defence in neighbouring plants at artificially high levels, physiologically relevant levels of VOCs appear instead to prime plants to prepare themselves for future pest and pathogen attack [10]. Priming is when plant defences are potentiated so that the response to subsequent attack is faster and stronger. Green-leaf volatiles (GLVs) prime *Z. mays* for enhanced production of the phytohormone jasmonic acid (JA) [11], and enhance expression of defence genes and metabolites in hybrid poplar [12]. Evidence of priming of direct and indirect defence in *Z. mays*, following exposure to *Spodoptera litteralis* induced-VOCs, was reported [13]. Exposure to VOCs from caterpillar-infested plants primed a subset of defence-related genes for earlier and/or stronger induction upon subsequent defence elicitation. This priming for defence-related gene expression correlated with reduced caterpillar feeding and development. Furthermore, exposure to caterpillar-induced VOCs primed for enhanced emissions of aromatic and terpenoid compounds. At the peak of this VOC emission, primed plants were significantly more attractive to the beneficial parasitic wasp *Cotesia marginiventris*. This study showed that VOC-induced priming targets a specific subset of JA-inducible genes, and linked these responses at the molecular level to enhanced levels of direct and indirect resistance against insect attack [13].

*cis*-Jasmone (CJ) is an oxylipin, produced naturally by plants, that was identified in our laboratory as an activator of plant defence, particularly in inducing resistance mechanisms in a manner different to, and potentially more valuable than, JA because of its ability to upregulate a specific set of defence genes [14], [15]. Since the initial discovery of CJ induced production of defence-related VOCs, including (*E*)-ocimene, in bean plants, *Vicia faba* [14], its effect on a number of other crop plants has been shown. Wheat plants, *Triticum aestivum*, when exposed to CJ, increased emission of (*E*)-ocimene, (*E*)-(1*R*,9*S*)-caryophyllene and 6-methyl-5-hepten-2-one, all important semiochemicals in tritrophic interactions, the latter of which is directly associated with attraction of parasitoids of aphid pests of wheat [16]. The increased production of these semiochemicals had a direct effect on settling of grain aphids, *Sitobion avenae*, numbers of which were significantly reduced on treated wheat compared to control plants in field simulation trials [17]. Furthermore, in laboratory development studies, the Mean Relative Growth Rate (MRGR) and the intrinsic rate of population increase (*r_m_*) were significantly reduced for *S. avenae* on treated seedlings compared to untreated seedlings [17]. In the field, replicated experiments conducted over four seasons demonstrated that winter wheat plots treated with CJ showed significantly reduced cereal aphid populations when compared to untreated control plots [17]. This was attributed to enhanced levels of allelopathic benzoxazinoids and phenolic acids [18]. CJ has recently been shown to induce indirect defence in soybean, *Glycine max*, leading to enhanced attraction of egg parasitoid natural enemies of stinkbug pests [19], and also to induce defence in cotton, *Gossypium hirsutum*, leading to repulsion of cotton aphids, *Aphis gossypii* [20]. For maize, however, there are as yet no reports on the impact of CJ, with studies of jasmonate-induced defence being restricted to JA [21], [22]. JA has been shown to induce production of defensive VOCs which cause repellency to herbivores [23] and antibiotic compounds which can reduce herbivore development [24]. However, JA, along with its volatile derivative methyl jasmonate (MJ), has been shown to have detrimental effects upon plants through the switching on of non-target genes [14], causing phytotoxicity and thereby minimising potential for their use in crop protection.

In view of our earlier studies showing a role for CJ in inducing indirect and direct defence in *Triticum aestivum*, another poaceous plant [16], [17], [18], and the previously reported priming of a specific subset of JA-inducible pathways in maize by defence VOCs [13], we investigated the ability of CJ to induce defence pathways in maize and to prime maize for enhanced defence against the leafhopper *C. storeyi*.

## Results

### Behavioural responses to CJ and CJ-treated plants

In a Y-tube olfactometer bioassay, the response of adult *C. storeyi* to CJ itself was not significantly different from a diethyl ether solvent control (*P*>0.05), at a dose of either 1 or 10 µg (Table 1). Furthermore, there was no significant difference in the amount of time spent by adult *C. storeyi* in the region of the olfactometer containing volatile organic compounds (VOCs) collected from CJ-treated maize seedlings, compared with the solvent control (*P*>0.05) or VOCs collected from untreated seedlings (*P*>0.05) (Table 1).

**Table 1:** Behavioural response (mean number ± SE) of *Cicadulina storeyi* leafhoppers to *cis*-jasmone (CJ), and VOCs collected from CJ-treated and untreated maize (*Zea mays*) seedlings in a Y-tube olfactometer.

Experiment	Treatment arm	Control arm	*P*
CJ (1 µg) .v. solvent (hexane) control	4.9±0.51	5.1±0.50	NS
CJ (10 µg) .v. solvent (hexane) control	4.7±0.26	5.3±0.26	NS
CJ-treated maize .v. untreated maize (control)	4.8±0.25	5.2±0.25	NS
CJ-treated maize .v. solvent (diethyl ether) control	4.8±0.49	5.2±0.49	NS

Data were analysed using a paired *t*-test. NS indicates no significant difference (*P*>0.05) between stimuli.

n = 10.

### Behavioural responses to CJ-treated plants exposed subsequently to *C. storeyi*

**Figure 1:**
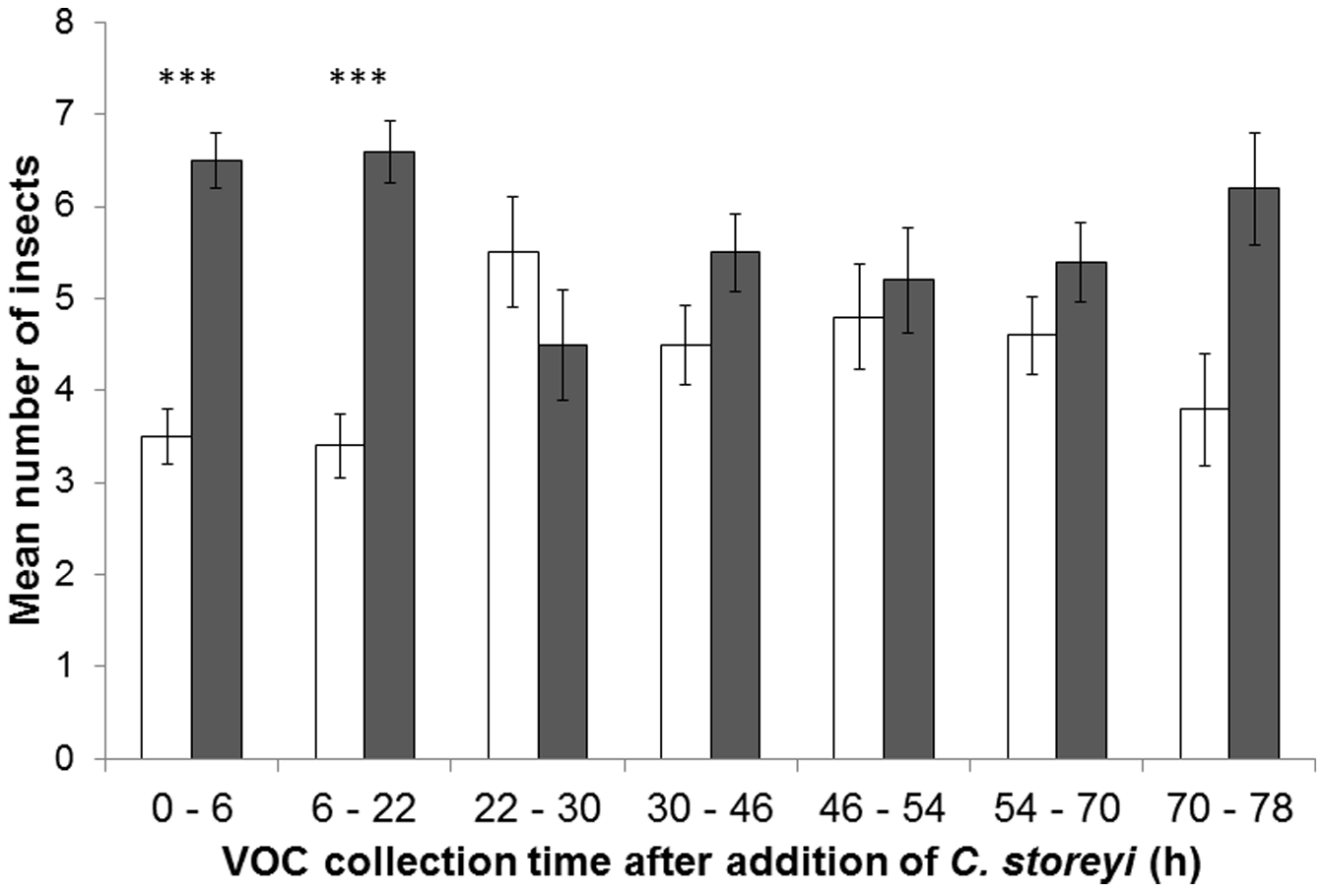
Behavioural response (mean number± SE) of *Cicadulina storeyi* adult leafhoppers to VOCs collected from *cis*-jasmone (CJ)-treated, adult *C. storeyi*-infested maize (*Zea mays*) seedlings versus VOCs collected from control-treated, adult *C. storeyi*-infested maize seedlings in a Y-tube olfactometer. VOCs were collected 0–3 h, 3–6 h, 6–22 h, 22–30 h, 30–46 h, 46–54 h, 54–70 h and 70–78 h after addition of *C. storeyi*. n = 10. Data were analysed using a paired *t*-test. ***P<0.001. White columns = control treatment, then *C. storeyi* infestation. Grey columns = CJ treatment, then *C. storeyi* infestation.

In a time course series of VOC collections from CJ- treated and control-treated seedlings subsequently infested with *C. storeyi*, followed by dual choice Y-tube olfactometer bioassays, adult *C. storeyi* significantly preferred VOCs from control-treated infested seedlings compared to VOCs from CJ-treated infested seedlings, collected 0–6 h and 6–22 h after *C. storeyi* adult infestation (Figure 1).

### Identification of VOCs emitted by CJ-treated plants exposed subsequently to *C. storeyi*

Treatment of maize seedlings with CJ followed by exposure to either *C. storeyi* adults or nymphs led to a significant increase in the emission of sesquiterpenes (Table 2). Seedlings treated with CJ produced significantly higher amounts of (*E*)-α-bergamotene and (*E*)-β-farnesene in the 0–6 h period after either *C. storeyi* adult or nymph infestation. Seedlings treated with CJ produced significantly higher amounts of (*E*)-(1*R*,9*S*)-caryophyllene and the homoterpene (*E*)-4,8-dimethyl-1,3,7-nonatriene (DMNT) in the 0–6 h period only after *C. storeyi* nymph infestation.

**Table 2:** Mean quantities (log_10_ng) of sesquiterpene VOCs and DMNT emitted by maize (*Zea mays*) seedlings following either *cis*-jasmone (CJ) or control treatment and then addition of *Cicadulina storeyi* (adults, n = 9 or nymphs, n = 10) 24 h later.

Time (h)	Treatment	(*E*)-(1*R*, 9*S*)-Caryophyllene		(*E*)-α-Bergamotene		(*E*)-β-Farnesene		Total sesquiterpenes		DMNT	
		Adults	Nymphs	Adults	Nymphs	Adults	Nymphs	Adults	Nymphs	Adults	Nymphs
0–6	CJ	3.157	2.091^a^	3.387^a^	1.885^a^	4.420^a^	2.662^a^	4.935^a^	3.444^a^	3.553	2.095^a^
	Control	2.219	0.977^b^	2.155^b^	0.658^b^	3.130^b^	1.653^b^	3.730^b^	2.334^b^	2.499	1.035^b^
6–22	CJ	5.180	4.175	5.450	4.327	6.200	5.145	6.818	5.769	3.341	3.063
	Control	5.003	4.233	4.956	4.199	5.781	5.046	6.430	5.729	3.186	3.333
22–30	CJ	5.010	4.297	4.960	4.195	5.777	5.152	6.440	5.780	3.072	3.172
	Control	4.716	4.208	4.550	3.928	5.381	4.971	6.081	5.602	2.929	2.801
30–46	CJ	5.710	4.967	5.111	4.542	5.883	5.448	6.739	6.181	3.107	3.352
	Control	5.897	4.865	5.164	4.335	5.985	5.292	6.876	6.038	3.215	2.853
46–54	CJ	4.857	4.401	3.999	3.793	4.914	4.743	5.810	5.515	2.537	2.775
	Control	4.869	4.180	3.953	3.501	4.785	4.541	5.739	5.297	2.665	2.397
54–70	CJ	5.584	4.955	4.392	4.087	5.298	5.008	6.362	5.893	2.842	3.322
	Control	5.336	4.655	4.106	3.619	5.018	4.580	6.063	5.516	3.009	2.815
70–78	CJ	4.691	4.364	3.504	3.468	4.449	5.540	5.533	5.384	2.364	2.450
	Control	4.880	4.390	3.605	3.475	4.442	4.548	5.582	5.374	2.861	2.772
Time by Treatment P-value		0.083	0.125	0.064	0.119	0.086	0.323	0.044	0.163	0.076	0.065
LSD1* (5%)		1.187	0.816	1.040	0.863	1.171	0.891	1.106	0.844	1.0913	0.904
LSD2** (5%)		0.553	0.609	0.633	0.610	0.659	0.633	0.589	0.600	0.6886	0.664

VOCs were collected after *C. storeyi* addition during the following time periods: 0–6 h, 6–22 h, 22–30 h, 30–46 h, 46–54 h, 54–70 h and 70–78 h. Data were analysed using a split-plot in time ANOVA. Appropriate means were compared using LSD (5%) values.

* For comparisons of treatments with or without CJ.

** For all other comparisons. For the 0–6 h collection period, means with superscript letters a and b within the same column are significantly different (*P*<0.05 LSD).

### Identification of VOCs emitted by CJ-treated plants exposed subsequently to JA

**Figure 2:**
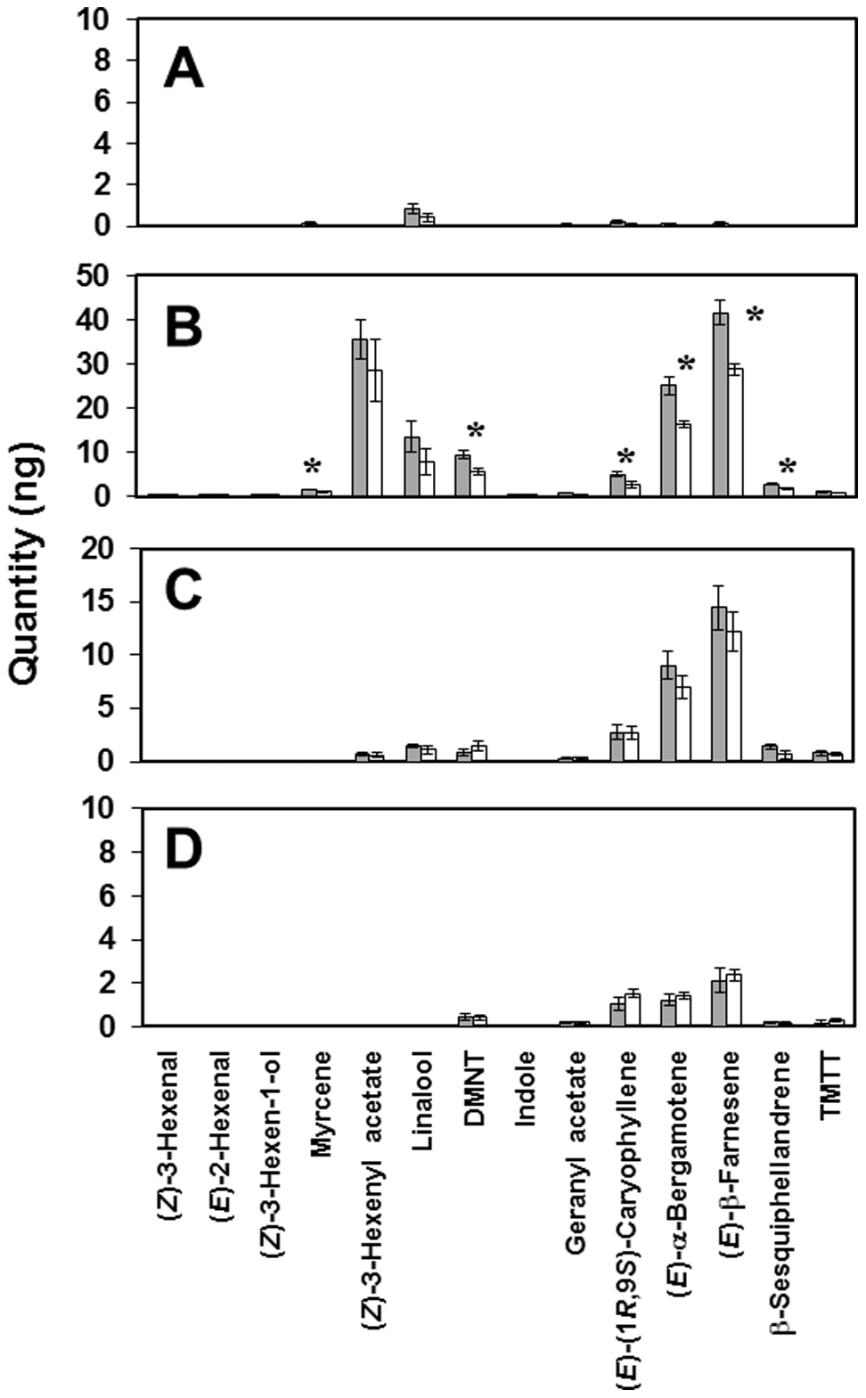
Mean quantities (± SE) of VOCs released by maize(*Zea mays*) seedlings following either CJ or control treatment, and then wounding+JA exposure 24 h later. VOCs were collected every 24 h over a continuous 96 h period. A = 24 h period between CJ or control treatment and wounding+JA exposure; B = 0–24 h after wounding+JA exposure; C = 24–48 h after wounding+JA exposure; D = 48–72 h after wounding+JA exposure. (*E*)-4,8-dimethyl-1,3,7-nonatriene = DMNT; (*E,E*)-4,8,12-dimethyl-1,3,7,11-tridecatetraene = TMTT. Data were expressed as nanograms of VOC released per 24 h collection period (9 plants were used per treatment) and were analysed using paired *t*-tests *P<0.05. n = 4. Grey columns = CJ treatment, then wounding+JA. White columns = control treatment, then wounding+JA.

To standardise the post-priming stimulus, a set amount of JA was used in initial VOC collections. GC analysis of VOCs collected from maize seedlings exposed to CJ, followed by wounding+JA treatment, showed there was considerable variation in emission of VOCs across the time points (Figure 2 A–D). In the first collection period after wounding+JA treatment (0–24 h, Figure 2B), emission of the monoterpene myrcene, DMNT, (*E*)-(1*R*,9*S*)-caryophyllene, (*E*)-α-bergamotene, (*E*)-β-farnesene and β-sesquiphellandrene was significantly greater from CJ-treated seedlings (*P*<0.05) compared to control-treated seedlings (Figure 2). No significant difference in VOC emission was observed between CJ-treated seedlings and control-treated seedlings pre-wounding, or 24–48 h and 48–72 h after wounding+JA.

## Discussion

In our previous chemical ecology study of the interaction between maize and *C. storeyi* leafhoppers, VOCs collected from uninfested maize seedlings were attractive, whilst VOCs collected from *C. storeyi*-infested seedlings were significantly repellent [5]. Furthermore, a number of compounds, ie. GLVs, aromatic compounds and isoprenoids, were shown to be released from infested seedlings, with sesquiterpenes and homoterpenes being either outright repellents or shown to interfere with attraction to host volatiles [5]. In the current study, we used behavioural and chemical approaches to investigate the potential for using the natural plant activator CJ to activate defence pathways in maize. Olfactometer bioassays using CJ-treated seedlings showed that CJ did not directly induce defence VOC production in maize. However, treatment of maize seedlings with CJ boosted production of volatile defence isoprenoids following subsequent addition of either *C. storeyi* adults or nymphs, or wounding+JA treatment. VOCs collected from CJ-treated, *C. storeyi*-infested seedlings were significantly more repellent than VOCs collected from control-treated, *C. storeyi*-infested seedlings. To our knowledge, this is the first experimental evidence demonstrating that CJ can prime plants for production of defence VOCs antagonistic to colonising herbivores.

The production of isoprenoids in plants is via terpene synthase (TPS) enzymes [25] and our results suggest that their activity is enhanced by priming maize seedlings with CJ. Many sesquiterpene synthase genes characterised to date are regulated by JA. For maize, these include TPS23 and TPS4, which are involved in (*E*)-(1*R*,9*S*)-caryophyllene and (*E*)-β-farnesene biosynthesis respectively. Effects on insects of the sesquiterpenes shown here to be primed by CJ are well known; for example, an (*E*)-β-farnesene synthase gene has been heterologously expressed in *Arabidopsis thaliana*, leading to repellency of aphids and recruitment of aphid parasitoids [26], whilst DMNT is released by maize to provide defence against stemborer moths [27]. Production of DMNT is a two-step process, initially involving production of (*E*)-nerolidol, followed by an oxidative cleavage. The first step in *A. thaliana* involves TPS04/GES, a TPS gene encoding a nerolidol/geranyllinalool synthase [28], whilst the second step involves CYP82G1, a cytochrome P450 monoxygenase, which catalyses the oxidative cleavage [29].

The results in our study suggest that CJ has the potential to augment induction of genes encoding terpene synthases in maize following herbivore damage. This extends previous work on VOC priming of defence in maize and raises the possibility of achieving this as a means of crop protection. VOC-induced priming has been shown to target defence pathways that are under JA control [11], and exposure of maize to VOCs from *Spodoptera littoralis* caterpillar-infested maize enhances induction of six caterpillar-inducible genes, which encode proteins with functions related to plant defence [13]. In the latter study, it was proposed that the enhanced induction of these genes could be used as markers for primed defence expression in maize. The results from our study suggest that CJ also has the ability to prime inducible pathways under the control of JA, despite CJ having an independent signalling role from JA [15]. However, the study of gene expression in maize following CJ treatment and subsequent exposure to JA or *C. storeyi* was outside the scope of this investigation. Further studies are planned to confirm the expression pattern of JA/*C. storeyi*-inducible genes in maize following CJ priming.

Analysis of the VOCs collected from CJ-treated, *C. storeyi*-infested seedlings and control- treated, *C. storeyi*-infested seedlings showed that the boosting of volatile sesquiterpene production was a temporary phenomenon. Seedlings treated with CJ and then exposed to wounding+JA produced enhanced levels of defence VOCs 0–24 h after wounding+JA treatment, and seedlings treated with CJ and then subjected to *C. storeyi* infestation produced enhanced levels of sesquiterpenes 0–6 h and 6–22 h after addition of *C. storeyi*. Beyond these timepoints, no significant differences in VOC levels were observed between CJ pre-treated and control pre-treated seedlings. This is to be expected because the JA or insect treatment would have induced VOC changes in unprimed plants after 24 h [5].

The priming effects we discovered are similar to those reported previously for VOC-induced priming against *S. littoralis* [13], in which enhanced levels coincided with improved attraction of *C. marginiventris*. Similar priming for indirect defence was also reported in lima beans [30], in which exposure to an artificial blend of VOCs resembling the blend from herbivore-infested plants led to enhanced production of extrafloral nectar upon wounding, which was attractive to predatory and parasitic insects. Both examples strongly suggest that VOC-induced priming is an ecologically important process, in which neighbouring intact plants are prepared for herbivore attack by response to defence VOCs released from nearby damaged plants.

As CJ is produced naturally by uninfested and herbivore-damaged plants (Oluwafemi and Birkett, unpublished data, [31]), this demonstration of enhanced defence VOC production upon herbivore damage following CJ treatment suggests that its natural release from herbivore-damaged plants can prime neighbouring plants for defence, and is therefore an important ecological event. Priming of plant defence signaling under ecologically realistic conditions has been reported previously [32], in which release of VOCs by sagebrush led to enhancement in neighbouring tobacco herbivore-regulated genes. An accelerated production of trypsin proteinase inhibitors was observed when *Manduca sexta* caterpillars fed on plants previously exposed to clipped sagebrush. The ability of CJ to prime maize for enhanced indirect defence through modification of natural enemy behaviour was outside the scope of this study, but laboratory and field behaviour investigations will be undertaken to confirm this.

There is increasing interest in understanding the underlying mechanisms of induced plant defence with a view to exploitation in novel sustainable pest control in agriculture, particularly as it can be exploited to provide targeted biological control through enhanced natural enemy foraging behaviour in conservation biological control [33]. Furthermore, constitutively expressed plant defence can be viewed as costly for plants, potentially leading to reduced crop yields [7]. Several studies have shown that volatile stress signalling from plants can prime recipient plants for direct and indirect defence [11], [13], , and can modify interactions with higher trophic levels. Our investigations on the activation of defence in crop plants show that CJ has the potential to be used as a novel crop protection agent through induction of indirect and direct defence [17], [18], [19], [20]. The results in this study highlight, for the first time, that CJ can also prime defence pathways in plants. Future work will investigate the potential for exploiting the priming phenomenon in field trials in maize.

## Materials and Methods

### Plants and leafhoppers

Maize (*Zea mays* cv. Delprim) seeds (Delley Samen und Pflanzen AG, Delley Semences et Plantes SA, Le Chateau, Switzerland) were potted in soil (Rothamsted Prescription mix) and grown under controlled conditions (25±1°C, 16 ∶ 8 h L∶D). *Cicadulina storeyi* China (Homoptera: Cicadelidae) leafhoppers, from the mass rearing colony of the International Institute of Tropical Agriculture (IITA), Ibadan, Nigeria, were reared on pearl millet, *Pennisetum americanum* ( = *typhoides*), in a quarantine growth facility (23±2°C, 40% RH, 16 ∶ 8 h L∶D). Leafhoppers and host plants were placed in Plexi-glass cages (44 cm×44 cm×70 cm) with two netted openings (12 cm×12 cm) in the front, while a third netted opening (11 cm×11 cm) at the back was fitted with an electric fan for air circulation.

### CJ application

CJ (90%; Avocado, Lancaster, UK) was formulated as a 0.1% aqueous emulsion with the non-ionic surfactant Ethylan BV (EBV) (Acros, Manchester, U.K.) and applied to maize seedlings (10–12 day old) at a rate equivalent to 50 g ha^−1^ using a hydraulic nozzle (Lurmark 015-F110) at 1 ms^−1^. Control seedlings were similarly sprayed with a 0.1% aqueous emulsion of EBV. Treated maize seedlings were kept in a glasshouse 24 h before being used in priming experiments.

### Volatile collection and quantification

Volatile organic compounds (VOCs) were collected from 10–12 day-old maize seedlings by air entrainment (also referred to as dynamic headspace collection) following standard procedures [34]. Seedlings were individually confined in glass vessels (22 cm high×6.5 cm internal diameter), open at the bottom but closed at the top except for two collection ports (one for inlet and the other for outlet). The bottom was closed without pressure around the plant stem by using two semicircular aluminium plates with a hole in the centre to accommodate the stem. The plates were clipped to a flange on the open end of the glass vessel. Air, purified by passage through an activated charcoal filter, was pumped into the vessel through the inlet port at 700 ml min^−1^. Air was drawn out at 600 ml min^−1^ through Porapak Q (50 mg; Alltech, PA, USA) in 5 mm diameter glass tubes (Alltech Associates, Camforth, Lancashire, UK). The difference in flow rates created a slight positive pressure to ensure that unfiltered air did not enter the system, thus removing the need for an airtight seal around the stem. All connections were made with polytetrafluoroethylene (PTFE) tubing (Alltech Associates) and brass Swagelock fittings (North London Valve, London, UK). Glassware, metal plates and other equipment were washed with Teepol detergent (Herts County Supplies, Herts, UK) in an aqueous solution, acetone and distilled water, and then baked overnight at 180°C. Porapak Q tubes were conditioned before use by washing with redistilled diethyl ether (1 ml, Sigma Aldrich) and heated to 132°C for 2 h under a constant stream of nitrogen. VOCs were eluted from the Porapak Q with 750 µl freshly-distilled diethyl ether, and stored in a tightly capped vial at −20°C until required for chemical analysis and behavioural assays.

VOC samples required for CJ-treated bioassays were collected from untreated and CJ treated seedlings up to 72 h after treatment. For experiments where CJ-treated or control-treated seedlings were subsequently exposed to *C. storeyi*, pre-treated maize seedlings were infested with either second-fourth instar *C. storeyi* nymphs or adults (100 per seedling), 24 h after treatment. VOCs were collected at 0–3 h, 3–6 h, 6–22 h, 22–30 h, 30–46 h, 46–54 h, 54–70 h and 70–78 h after infestation was initiated. Nine and ten replicates were done respectively for adult and nymph infestation. For experiments where CJ-treated plants were subsequently exposed to wounding+JA, the undersides of two leaves of CJ or control-treated seedlings were scratched (1 cm^2^) on both sides of the central vein with a razor blade, 24 h after treatment. JA (250 µM in deionized water) was immediately applied evenly over the scratched areas using forceps, n = 4 [13]. VOCs were collected for 24 h prior to wounding+JA treatment, then at 24 h periods for up to 72 h afterwards.

VOC samples (4 µl) were analysed on a Hewlett-Packard 6890 GC equipped with a cool-on-column injector, a flame ionization detector (FID) and a non-polar HP-1 bonded phase fused silica capillary column (J & W Scientific, 50 m×0.32 mm i.d., 0.52 µm film thickness). The oven temperature was maintained at 30°C for 1 min, and programmed at 5°C min^−1^ to 150°C, then 10°C min^−1^ to 230°C and held for 20 min. The carrier gas was hydrogen. Quantification of VOCs identified by coupled gas chromatography-mass spectrometry (GC-MS) was performed using a single point internal [35] or external [36] standard quantification method using authentic samples of standards.

GC-MS analysis of VOC samples was performed using a fused silica capillary column (50 m×0.32 mm i.d., 0.52 µm film thickness, HP-1, J & W Scientific), attached to a cool on-column injector, which was directly coupled to a magnetic sector mass spectrometer (Autospec Ultima, Fisons Instruments, Manchester, UK). Ionization was by electron impact (70 eV, source temperature 250°C). Helium was the carrier gas. The oven temperature was maintained at 30°C for 5 min, and then programmed at 5°C min^−1^ to 250°C. Identifications were made by comparison of spectra with mass spectral databases [37], and comparison of retention times/indices with those from previous analyses which applied a fully rigorous identification procedure [5].

### Y-tube olfactometer bioassays

A glass Y-tube olfactometer (2 cm internal diameter, 16 cm stem length, 14 cm arm length) was clamped on a tripod in a black cage (60 cm×60 cm×76 cm, steel frame but covered with black cardboard paper) in an inclined position (70° from the horizontal plane) with two fluorescent light tubes (70 W; Luminux) positioned approximately 25 cm above the Y-tube junction. Air was pumped, using an electric pump, through an activated charcoal filter to remove chemical contaminants before being divided into two. Two flow meters were used to ensure that the air streams entered the two arms of the olfactometer at the same rate (200 ml min^−1^). Before entering the Y-tube, each airstream passed through a glass jar containing a filter paper strip onto which either the treatment or control solution was applied (1 µl). Leafhoppers were collected from the rearing cages and kept in batches within aspirators for two hours of fasting before each experiment. Ten adult leafhoppers were introduced to the Y-tube and used in each replicate, and experiments were replicated 10 times. After each replicate, the apparatus was rotated 180° to avoid position effects. A leafhopper was deemed to have made a final choice if it entered into an arm and stayed there for 10 min. The bioassays were conducted in a dark controlled environment room (22±1°C, 40% RH) fitted with an extraction fan. The following choice bioassay experiments were carried out: (1) CJ (1 µl of a 1 µg/µl solution in diethyl ether) versus diethyl ether solvent (1 µl); (2) CJ (1 µl of a 10 µg/µl solution in diethyl ether) versus diethyl ether solvent (1 µl); (3) VOC sample from CJ-treated maize seedlings (1 µl) versus VOC sample from untreated maize seedlings (1 µl); (4) VOC sample from CJ-treated maize seedlings (1 µl) versus diethyl ether (1 µl); (5) VOC sample from CJ-treated, *C. storeyi*-exposed seedlings (1 µl) versus VOC sample from control-treated, *C. storeyi*-exposed seedlings (1 µl), both samples being collected at the same time point, *ie.* 0–3 h, 3–6 h, 6–22 h, 22–30 h, 30–46 h, 46–54 h, 54–70 h and 70–78 h after *C. storeyi* infestation.

### Statistical analysis

The Y-tube olfactometer data were analysed using a paired *t*-test after ensuring that data were normally distributed. VOC data from CJ or control-treated seedlings exposed subsequently to *C. storeyi* were log transformed,and analysed using a split-plot in time analysis of variance (ANOVA) to assess the main effect of treatment (with or without CJ treatment), time, and the interaction of these two factors. Following ANOVA, appropriate means were compared using least significant difference (LSD) values at the 5% level of significance. VOC data from CJ or control-treated seedlings exposed subsequently to wounding+JA treatment were analysed using a paired *t*-test. Statistical analyses were done using the GenStat statistical system (GenStat® 2006, Tenth Edition © VSN International Ltd., Hemel Hempstead, UK).

## References

[1] Thottappilly G, Bosque-Perez NA, Rossel HW (1993) Viruses and virus diseases of maize in tropical Africa. Plant Pathol 42: 494–509.

[2] Asanzi MC, Bosque-Perez NA, Nault LR, Gordon DT, Thottappilly G (1995) Biology of *Cicadulina* species (Homoptera: Cicadellidae) and transmission of maize streak virus. African Entomol 3: 173–179.

[3] Oluwafemi S, Jackai LEN, Alegbejo MD (2007) Comparison of transmission abilities of four *Cicadulina* species vectors of maize streak virus from Nigeria. Entomol Exp Appl 124: 235–239.

[4] Kim SK, Efron Y, Singh J, Buddenhagen IW, Asnasi VL, et al. (1981) Recent progress on maize streak resistant breeding program at IITA. Paper 3^rd^ OAU/STRC Workshop on maize and cowpea held at IITA, Ibadan, Nigeria. Feb 23–27th.

[5] Oluwafemi S, Bruce TJA, Pickett JA, Ton J, Birkett MA (2011) Behavioural Responses of the Leafhopper, *Cicadulina storeyi* China, a Major Vector of Maize Streak Virus, to Volatile Cues from Intact and Leafhopper-Damaged Maize. J Chem Ecol 37: 40–48. 21191806 10.1007/s10886-010-9891-2

[6] Turlings TCJ, Ton J (2006) Exploiting scents of distress: the prospect of manipulating herbivore-induced plant odors to enhance the control of agricultural pests. Curr Opin Plant Biol 9: 421–427. 16723271 10.1016/j.pbi.2006.05.010

[7] Bruce TJA, Pickett JA (2007) Plant defence signalling induced by biotic attacks. Curr Opin Plant Biol 10: 387–392. 17627867 10.1016/j.pbi.2007.05.002

[8] Chamberlain KC, Pickett JA, Woodcock CM (2000) Plant signalling and induced defence in insect attack. Mol Plant Pathol 1: 67–72. 20572952 10.1046/j.1364-3703.2000.00009.x

[9] Pickett JA, Poppy GM (2001) Switching on plant genes by external chemical signals. Trends Plant Sci 6: 137–139. 11286900 10.1016/s1360-1385(01)01899-4

[10] Heil M, Ton J (2008) Long distance signalling in plant defence. Trends Plant Sci 13: 264–272. 18487073 10.1016/j.tplants.2008.03.005

[11] Engelberth J, Alborn HT, Schmelz EA, Tumlinson JH (2004) Airborne signals prime plants against insect herbivore attack. Proc Natl Acad Sci USA 101: 1781–1785. 14749516 10.1073/pnas.0308037100 PMC341853

[12] Frost CJ, Mescher MC, Dervinis C, Davis JM, Carlson JE, et al (2008) Priming defense genes and metabolites in hybrid polar by the green leaf volatile *cis*-3-hexenyl acetate. New Phytol 180: 722–734. 18721163 10.1111/j.1469-8137.2008.02599.x

[13] Ton J, D'Alessandro M, Jourdie V, Jakab G, Karlen D, et al (2006) Priming by airborne signals boosts direct and indirect resistance in maize. Plant J 49: 16–26. 17144894 10.1111/j.1365-313X.2006.02935.x

[14] Birkett MA, Campbell CAM, Chamberlain K, Guerrieri E, Hick AJ, et al (2000) New roles for *cis*-jasmone as an insect semiochemical and in plant defence against insects. Proc Natl Acad Sci USA 97: 9329–9334. 10900270 10.1073/pnas.160241697 PMC16867

[15] Bruce TJA, Matthes M, Chamberlain K, Woodcock CM, Mohib A, et al (2008) *cis*-Jasmone induces *Arabidopsis* genes that affect the chemical ecology of multitrophic interactions with aphids and their parasitoids. Proc Natl Acad Sci USA 105: 4553–4558. 18356298 10.1073/pnas.0710305105 PMC2290791

[16] Pickett JA, Birkett MA, Bruce TJ, Chamberlain K, Gordon-Weeks R, et al (2007) Developments in aspects of ecological phytochemistry: the role of *cis*-jasmone in inducible defence systems in plants. Phytochemistry 68: 2937–2945. 18023830 10.1016/j.phytochem.2007.09.025

[17] Bruce TJA, Martin JL, Pickett JA, Pye BJ, Smart LE, et al (2003) *cis*-Jasmone treatment induces resistance in wheat plants against the grain aphid, *Sitobion avenae* (Fabricius) (Homoptera: Aphididae). Pest Man Sci 59: 1031–1036. 10.1002/ps.730 12974355

[18] Moraes MCB, Birkett MA, Gordon-Weeks R, Smart LE, Martin JL, et al (2008) *cis*-Jasmone induces accumulation of defence compounds in wheat, *Triticum aestivum* . Phytochemistry 69: 9–17. 17681563 10.1016/j.phytochem.2007.06.020

[19] Moraes MCB, Sereno FTPS, Micheriff MFF, Pareja M, Laumann RA, et al (2009) Attraction of the stink bug egg parasitoid, *Telenomus podisi* (Hymenoptera: Scelionidae) to defence signals from soybean, *Glycine max* (Fabaceae), activated by treatment with *cis*-jasmone. Entomol Exp Appl 131: 178–188.

[20] Hegde M, Nobre Oliveira J, da Costa JG, Bleicher E, Goulart Santana AE, et al (2012) Aphid antixenosis in cotton is activated by the natural plant activator *cis*-jasmone. Phytochemistry 78: 81–88. 22516741 10.1016/j.phytochem.2012.03.004

[21] Schmelz EA, Alborn HT, Banchio E, Tumlinson JH (2003) Quantitative relationships between induced jasmonic acid levels and volatile emission in *Zea mays* during *Spodoptera exigua* herbivory. Planta 216: 665–673. 12569409 10.1007/s00425-002-0898-y

[22] Hopke J, Donath J, Blechert S, Boland W (1994) Herbivore-induced volatiles: the emission of acyclic homoterpenes from leaves of *Phaseous lunatus* and *Zea mays* can be triggered by a β-glucosidase and jasmonic acid. FEBS Lett 352: 146–150. 7925964 10.1016/0014-5793(94)00948-1

[23] Ozawa R, Shiojiri K, Sabelis MW, Arimura GI, Nishioka T, et al (2004) Corn plants treated with jasmonic acid attract more specialist parasitoids, thereby increasing parasitization of the common armyworm. J Chem Ecol 30: 1797–1808. 15586675 10.1023/b:joec.0000042402.04012.c7

[24] Oiwaka A, Ishihara A, Hasewaga M, Koduma O, Iwamura H (2001) Induced accumulation of 2-hydroxy-4,7-dimethoxy-1,4-benzoxazin-3-one glucoside (HDMBOA-Glc) in maize leaves. Phytochemistry 56: 669–675. 11314951 10.1016/s0031-9422(00)00494-5

[25] Degenhardt J, Kollner TG, Gershenzon J (2009) Monoterpene and sesquiterpene synthases and the origin of terpene skeletal diversity in plants. Phytochemistry 70: 1621–1637. 19793600 10.1016/j.phytochem.2009.07.030

[26] Beale MH, Birkett MA, Bruce TJ, Chamberlain K, Field LM, et al (2006) Aphid alarm pheromone produced by transgenic plants affects aphid and parasitoid behavior. Proc Natl Acad Sci USA 103: 10509–10513. 16798877 10.1073/pnas.0603998103 PMC1502488

[27] Tamiru A, Bruce T, Woodcock C, Caulfield J, Midega C, et al (2011) Maize landraces recruit egg and larval parasitoids in response to egg deposition by a herbivore. Ecol Lett 14: 1075–1083. 21831133 10.1111/j.1461-0248.2011.01674.x

[28] Herde M, Gärtner K, Köllner TG, Fode B, Boland W, et al (2008) Identification and Regulation of TPS04/GES, an *Arabidopsis* geranyllinalool synthase catalyzing the first step in the formation of the insect-induced volatile C_16_-homoterpene TMTT. Plant Cell 20: 1152–1168. 18398052 10.1105/tpc.106.049478 PMC2390743

[29] Lee S, Badieyan S, Bevan DR, Herde M, Gatz C, et al (2010) Herbivore-induced and floral homoterpene volatiles are biosynthesized by a single P450 enzyme (CYP82G1) in *Arabidopsis* . Proc Natl Acad Sci USA 107: 21205–21210. 21088219 10.1073/pnas.1009975107 PMC3000306

[30] Kost C, Heil M (2006) Herbivore-induced plant volatiles induce an indirect defence in neighbouring plants. J Ecology 94: 619–628.

[31] Loughrin JH, Manukian A, Heath RR, Turlings TCJ (1994) Diurnal cycle of emission of induced volatile terpenoids by herbivore-injured cotton plants. Proc Natl Acad Sci USA 91: 11836–11840. 11607499 10.1073/pnas.91.25.11836 PMC45330

[32] Kessler A, Halitschke R, Diezel C, Baldwin IT (2006) Priming of plant defence responses in nature by airborne signalling between *Artemesia tridentata* and *Nicotiana attenuata* . Oecologia 148: 280–292. 16463175 10.1007/s00442-006-0365-8

[33] Powell W, Pickett JA (2003) Manipulation of parasitoids for aphid pest management: Progress and prospects. Pest Man Sci 59: 149–155. 10.1002/ps.550 12587868

[34] Agelopoulos NG, Hooper AM, Maniar SP, Pickett JA, Wadhams LJ (1999) A novel approach for isolation of volatile chemicals released by individual leaves of a plant *in situ* . J Chem Ecol 25: 1411–1425.

[35] D'Alessandro M, Turlings TCJ (2005) In situ modification of herbivore-induced plant odors: a novel approach to study the attractiveness of volatile organic compounds to parasitic wasps. Chem Senses 30: 739–753. 16243967 10.1093/chemse/bji066

[36] Skelton AC, Cameron MM, Pickett JA, Birkett MA (2010) Identification of neryl formate as an airborne aggregation pheromone for the American house dust mite, *Dermatophagoides farinae*, and the European house dust mite, *Dermatophagoides pteronyssinus* (Acari: Epidermoptidae). J Med Entomol 47: 798–804. 20939374 10.1603/me09295

[37] NIST (2005) NIST mass spectral search for the NIST/EPA/NIH mass spectral library version 2.0. Office of the Standard Reference Data Base, National Institute of Standards and Technology, Gaithersburg, Maryland.

